# Evaluation of P53 protein expression in gingival tissues of patients with chronic periodontitis by immunohistochemistry methods

**DOI:** 10.1002/cre2.668

**Published:** 2022-10-20

**Authors:** Samaneh Minabian, Shima Soleimani S., Molook Torabi, Mohammad Mohammadi, Hadi Ranjbar

**Affiliations:** ^1^ Oral and Dental Diseases Research Center Kerman University of Medical Sciences Kerman Iran; ^2^ Oral and Maxillofacial Pathology Department, School of Dentistry Kerman University of Medical Sciences Kerman Iran; ^3^ Periodontics Department, School of Dentistry Kerman University of Medical Sciences Kerman Iran; ^4^ Mental Health Research Center, Psychological Health Research Institute Iran University of Medical Sciences Tehran Iran

**Keywords:** apoptosis, chronic periodontitis, expression, P53

## Abstract

**Objective:**

Periodontitis is one of the most important periodontal diseases that can be affected by many factors. Although the mechanism of periodontitis development is not yet fully understood, previous studies suggest that apoptosis may be one of the pathological factors that can affect the process of the disease by destroying old and damaged cells. Low expression of P53 protein is one of the reasons for delaying cell death that allows damaged cells to survive longer and gives more time for the chance of mutations and pathogenesis. Because of the important role of P53 in gingival cells of patients with chronic periodontitis, the objective of our study is to evaluate the P53 protein expression in gingival tissues of patients with chronic periodontitis by immunohistochemistry methods.

**Materials and Methods:**

In this cross‐sectional study, 35 patients with severe to moderate chronic periodontitis (loss of attachment ≥3 mm, probing depth ≥5 mm) with no treatment and 25 people who were healthy for periodontal problems were examined. Gingival biopsies from marginal and attached gingiva were obtained, prepared, and mounted on slides. Then, the expression of P53 on each slide was evaluated by optic microscopy after using P53 antibodies and staining with hematoxylin‐eosin (immunohistochemistry method). Data were analyzed using independent *t*‐test, Mann–Whitney U‐test, and Spearman correlation test using SPSS Statistics version 18.0.

**Results:**

The mean ages of participants in the case and control groups were 37.58 and 32.09, respectively. Our results showed that the expression of P53 was not significant in periodontitis compared to the control group (*p* > .05). Also, gender could not affect the expression of P53 in both groups (*p* > .05), and there was no significant relationship between age and P53 gene incidence.

**Conclusion:**

Chronic periodontitis has no significant effect on P53 expression, so changes in apoptosis due to P53 expression in periodontitis are not significant.

## 1 INTRODUCTION

Periodontal disease is an inflammatory disease of the periodontium and its advanced form is characterized by periodontal ligament loss and destruction of surrounding alveolar bone, which is the main cause of tooth loss (De Pablo et al., 2009). Periodontitis is one of the most common periodontal diseases. It is a multifactorial disease that can be affected by genetic, environmental factors, living standards, and systemic diseases (AlJehani, 2014; Cheng et al., 2015; De Pablo et al., 2009; Frías‐Muñoz et al., 2018; Yoon et al., 2017). Data from previous studies have shown the different prevalence of periodontitis in the population, which is ranged from 13.5% to 83.5% (Balaji et al., 2018; Machado et al., 2018; Singh et al., 2012; Susin et al., 2011; Wellapuli & Ekanayake, 2017). Also, some studies suggested that the risk of developing periodontitis increases with age and occurs in females more than in males (AlJehani, 2014; Robo et al., 2021).

One of the main causes of periodontal diseases is Gram‐negative organisms, so actinobacillus actinomycetemcomitans (aa), an anaerobic Gram‐negative rod, is considered to be one of the major etiological agents of chronic periodontitis (Loomer & Armitage, 2004). These bacteria cause the migration of neutrophils to the site of infection and result in the secretion of cytokines and inflammatory mediators, which seems to be effective in the development and progression of periodontal diseases. The mechanisms of periodontal tissue damage have not been completely elucidated, and previous studies suggest that both immune‐mediated reactions and direct cytopathic effects of bacteria may be involved (Bulut et al., 2006). Because of the direct effect of bacteria in cell cultures, it has been assumed that apoptosis might play an important role in periodontitis (Bulut et al., 2006). However, the nature of molecular mechanisms participating in this process remains unknown (Bulut et al., 2006). In addition, lipopolysaccharides (LPSs) in the cell wall of Gram‐negative bacteria stimulate butyric acid‐induced apoptosis in human peripheral blood mononuclear cells, and an aa toxin induces apoptosis in B lymphocytes present in periodontal tissues (Kurita‐Ochiai et al., 1997, 1999; Ohguchi et al., 1998). According to the literature, it can be concluded that apoptotic mechanisms play an important role in the pathogenesis of periodontal diseases, including chronic periodontitis.

Apoptosis is the cell's natural mechanism for programmed cell death. It is a highly regulated process that eliminates unnecessary or dangerous cells (Danial & Korsmeyer, 2004; Lopez & Tait, 2015). The process of apoptosis can be altered by stimuli such as hormones, cytokines, growth factors, bacterial and viral infections, and immune responses (Thompson, 1995). Understanding apoptosis in disease conditions is vital because it can determine the disease pathogenesis and assist clinicians in finding the best treatment approach. In cancer, the balance between cell division and cell death is disturbed and cells that should have died did not receive death signals. This problem can occur at any stage of the apoptotic pathway (Bauer & Helfand, 2006). One of the reasons for delayed cell death is the downregulation of P53, a member of the Bcl‐2 family that encodes P53 protein and is a tumor suppressor. P53 controls the cell cycle and activates apoptosis. Mutation in the P53 gene results in reduced apoptosis and the loss of apoptotic control allows cancer cells to survive longer and gives more time for the chance of mutations. These can increase invasiveness during tumor progression, stimulate angiogenesis, deregulate cell proliferation, and interfere with differentiation (Bauer & Helfand, 2006; Gasco et al., 2002; Hassan et al., 2014; Morton et al., 2010; Rodrigues et al., 1990).

While Bulut et al. 's (2006) study showed that there was no significant difference in P53 expression in the gingival cells of people with aggressive periodontitis and the control group, another study found a slight increase in P53 levels in people with aggressive periodontitis compared to healthy people (Bullon et al., 2004). On the other hand, some recent studies had reported that although the level of P53 cannot be detected in healthy tissues, this rate has increased significantly in the gingival epithelial cells of people with periodontitis and can be detected by immunohistochemistry methods (Kasprzak et al., 2014; Memmert et al., 2016).

Since previous studies indicate controversial results, and the expression of P53 protein in gingival cells of patients who have chronic periodontitis is still poorly understood, this study aims to evaluate the P53 protein expression in gingival tissues of patients with chronic periodontitis. The null hypothesis predicts no changes in the expression of P53 protein in gingival tissues of chronic periodontal patients compared to healthy people.

## 2 MATERIALS AND METHODS

### 2.1 Study population

In this cross‐sectional study, patients with moderate to severe chronic periodontitis (loss of attachment ≥3 mm and probing depth ≥5 mm) who were not treated were evaluated and formed the case group. The control group included healthy people without neither systemic diseases nor periodontal diseases. The members of both groups were chosen from people with confirmed gingival or any oral surgery who were referred to the Department of Periodontology, School of Dentistry, Kerman, Iran. The convenience sampling method was used, and the sample size was calculated using G*Power with the following parameters (power of test = 0.8, level of significance *α* = .05, effect size = 0.82). The minimum sample size for each group was determined *n* = 25. Written consent was obtained from all individuals for this study. This study was approved by the Ethics Committee of Kerman University of Medical Science (permission number: IR.KMU.REC12/93/209).

### 2.2 Inclusion and exclusion criteria

Inclusion criteria for the participants were as follows: (1) subjects with at least 14 teeth and 10 posterior teeth; (2) no use of drugs affecting the periodontium, such as Phenytoin and Nifedipine; (3) no use of nonsteroid anti‐inflammatory drugs (NSAIDs) and antibiotics during the last 6 months; (4) no use of cigarettes, alcohol, and other opiates; (5) no history of cancer; (6) no history of chemotherapy and radiotherapy; (7) no family history of cancer; (8) no systemic diseases; and (9) consent to participate in the study.

### 2.3 Sample preparation method

A biopsy of approximately 3 × 2 mm was taken from the marginal or attached gingiva in all participants using a scalpel‐15 and was kept in 10% formalin. Biopsies were transferred to the pathology laboratory of the dental school, and after the preparation of paraffin blocks, these blocks were sent to Afzalipour hospital's pathology laboratory. Immunohistochemistry of P53 protein was performed as follows:

(1) The 10‐micron sections were prepared from paraffin blocks of tissue biopsies and were mounted on slides. (2) The sections were incubated in buffer citrate (10 mmol for 20 min) in the microwave. (3) Then, sections were incubated in 0.5% hydrogen peroxide for about 15–20 min. (4) Sections were covered with 2% bovine serum albumin (15 min). (5) The sections were then incubated overnight with a specific P53 antibody; the dilution of each antibody was determined according to the initial tests. (6) Sections were rinsed with the second antibody, conjugated to biotin, for 30 min. (7) If required, sections were washed with phosphate‐buffered saline (PBS) three times in each step. (8) Sections were incubated with 3,3’‐diaminobenzidine (DAB) chromogenic (5–10 min) stain. (9) At the end, sections were washed with distilled water three times (5 min) and stained with hematoxylin–eosin (10 s). (10) After washing, they were covered with special immunocytochemistry glue. 11. The sections were kept for imaging and data collection.

A pathologist who was blinded to the study (a code was given to each slide) evaluated the slides. The results of immunohistochemistry methods were evaluated by dividing the average percentage of positive cells (stained cells) by the total number of cells (positive and negative cells) in at least three microscopic fields using an optical microscope with X400 magnification (Bulut et al., 2006).

To evaluate the expression of the P53 protein, we used the following criteria:

– Staining of less than 25% of the counted cells.
– Staining of 25%–50% of the counted cells.
– Staining of 50%–75% of the counted cells.
– Staining of 75%–100% of the counted cells (Melcher, 1976).

### 2.4 Statistical analysis

The data were analyzed using independent *t*‐test, Mann–Whitney U‐test, and Spearman correlation test, and the statistical analysis was performed using SPSS Statistics version 18.0 [“IBM SPSS Statistics for Windows.” IBM Corp., 2011.]. Moreover, we considered a *p*‐value of less than 0.05 statistically significant.

## 3 RESULTS

**Figure 1:**
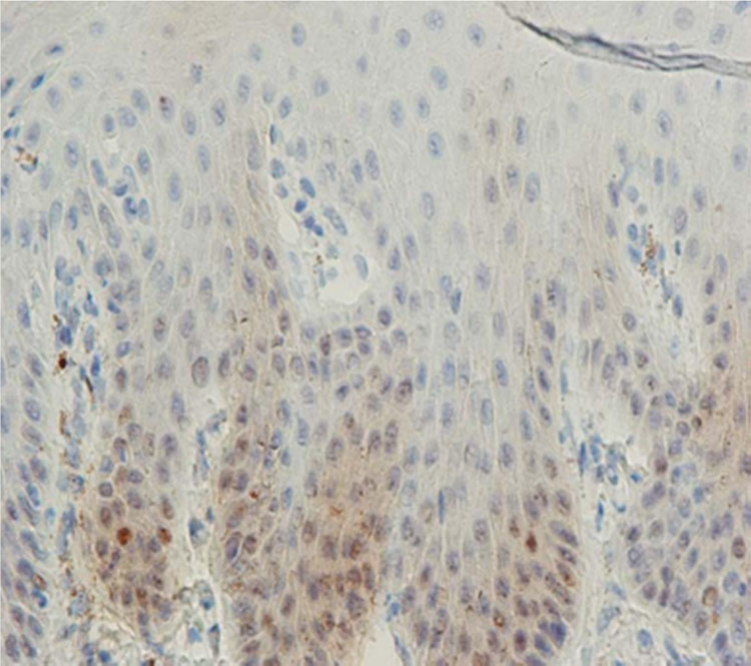
Expression of P53 in the gingival tissue

**Figure 2:**
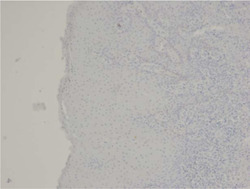
Lack of expression of P53 in the gingival tissue

In this study, a total number of 35 patients (19 men and 16 women) with a mean age of 37.58 (men = 38.05, female = 37.12) in the periodontitis group and 25 patients (7 men and 18 women) with a mean age of 32.09 (men = 30.57, female = 33.61) in the control group were evaluated for staining percentage of gingival epithelial cells with the P53 marker. In our study, there was no significant difference in the mean age of men and women in both control and periodontitis groups (*p* > .05). The results of this study showed no statistically significant difference in the expression of P53 in gingival epithelial cells of patients with periodontitis compared to the control group (*p* > .05) (Table 1, Figures 1 and 2).

**Table 1:** Staining percentage of gingival epithelial cells with the P53 marker in periodontitis and control groups

Groups	% P53 expression (mean ± SD)	*p*‐Value
Periodontitis (*n* = 35)	0.54 ± 1.65	.36
Control (*n* = 25)	0.20 ± 1.00	

As shown in Table 2, the results reported that gender has no significant effect on the staining percentage of epithelial cells in both groups (comparison of men with women) (*p* > .05). Furthermore, the findings indicated that there was no statistically significant difference between men in the control group and men in the periodontitis group in terms of the staining percentage of gingival epithelial cells with the P53 marker; moreover, in the comparison between the women of the two groups, the result was the same (*p* > .05) (Table 2).

**Table 2:** Staining percentage of gingival epithelial cells with the P53 marker and its relationship with gender in periodontitis and control groups

Groups		% P53 expression (mean ± SD)	*p*‐Value
Periodontitis (*n* = 35)	Male (*n* = 19)	0.47 ± 0.96	.68
	Female (*n* = 16)	0.62 ± 2.24	
Control (*n* = 25)	Male (*n* = 7)	0	.42
	Female (*n* = 18)	0.27 ± 1.17	
Male	Periodontitis (*n* = 19)	0.47 ± 0.96	.21
	Control male (*n* = 7)	0	
Female	Periodontitis (*n* = 16)	0.62 ± 2.24	.74
	Control (*n* = 18)	0.27 ± 1.17	

The Spearman correlation test showed that there was no statistically significant relationship between age and incidence of the P53 gene. The results of this correlation were rho = .06, *p* = .73 for the periodontitis group and rho = .34, *p* = .09 for the control group.

## 4 DISCUSSION

The present study was designed to determine the effect of periodontitis on the expression of P53 protein in gingival tissues by using immunohistochemistry methods. As P53 plays an important role in tumor suppression by controlling the apoptosis process (Morton et al., 2010; Rodrigues et al., 1990), it is necessary for clinicians to know its cellular regulation in patients to properly manage and treat its associated complications. Since previous studies have been of little agreement on the expression changes of P53 in gingival tissues of patients with periodontitis (Aral et al., 2019; Bullon et al., 2004; Bulut et al., 2006; Gamonal et al., 2001), we conducted this study to shed more light on the subject.

We found that there was no significant difference in the expression of P53 between periodontitis and the control groups. This accords with the earlier observation by Bulut et al. (2006), which reported that the expressions of P53 and Caspase‐3 have shown no significant difference between periodontitis and the control group, but there was an increase in the expression rate of Bcl‐2 in patients with periodontitis. Although the methods of both studies were almost similar, in the present study, we extended the number of participants to 35 patients with chronic periodontitis. This increase in sample size in our study partially compensates for the weakness of the study by Bulut et al. (2006), which examined only eight patients. Gamonal et al. (2001) evaluated the expression of P53, Bcl‐2, Fas/Fasl, and Caspase‐3 in gingival tissues of patients with periodontitis and subjects who had healthy periodontium as the control group by immunohistochemistry methods. They showed the presence of apoptotic cells in biopsy samples provided from areas with probing depth ≥ 5 mm and attachment loss ≥ 3 mm, which strongly supports the existence of the apoptosis process in patients with chronic periodontitis. Furthermore, their study concluded that the expression of P53 protein and Bcl‐2 increased in chronic periodontitis. However, this observation is not consistent with the results of our study, where no significant difference in the expression of P53 between the periodontitis group and the control group was found. A possible explanation might be that in Gamonal et al. 's study an electron microscope was used to evaluate the results of the immunohistochemistry method, but we used an optic microscope, which can be considered as a limitation in the present study. Another potential limitation is that we did not include demographic variables (except for sex and age) in statistical analysis, although previous studies have not investigated this information either (Bulut et al., 2006; Gamonal et al., 2001). In addition, periodontal parameters such as bleeding and gingival index were not evaluated in our study.

On the other hand, Bullon et al. (2004) compared some apoptotic markers in peri‐implantitis cases and healthy peri‐implant cases and concluded that epithelial cells were negative for bcl‐2 in the case of peri‐implantitis and showed a faint expression of P53 protein in the parabasal layer. In a study that set out to determine the clinical outcomes of nonsurgical periodontal treatment with antibiotics on apoptosis markers in aggressive periodontitis, Aral et al. (2019) found that their treatment approach decreased the P53 level for 6 months.

Another important finding of our study is that no significant difference between the percentage of staining of gingival epithelial cells and gender in periodontitis and control groups was observed (comparison of men with women). In addition, the results reported that there was no statistically significant difference in the comparison of men in the control group with men in the periodontitis group in terms of the staining percentage of gingival epithelial cells with the P53 marker. Similarly, in the same comparison between the women, the difference was not significant too. To our knowledge, this is the first study that evaluated the effect of sex on P53 expression.

About 150 years ago, Virtue first stated that there is an undeniable link between inflammation and cancer, as inflammatory cells are present in tumors and tumors arise at sites of chronic inflammation. These observations concluded that inflammation significantly helps in the development of cancer. Recent evidence support this conclusion and estimate that up to 25% of all cancers are due to chronic infection or other types of chronic inflammation (Perwez Hussain & Harris, 2007). All of these suggest that a well‐regulated inflammatory response can have a role in tumor suppression (Mantovani et al., 2008). Chronic inflammation such as periodontitis, however, is injurious and can predispose cells for an oncogenic transformation and alter the expression of oncogenes and tumor suppressor genes to promote neoplastic transformation (Schetter et al., 2009). P53 is a tumor suppressor that is often mutated in malignancies whose function is to regulate important cellular activities such as cell cycle, aging, and apoptosis. Because inflammation and cancer are strongly linked through common pathways, P53 can suppress inflammation in human tissues through one of two different routes: cell cycle arrest or apoptotic cell death (Barabutis et al., 2018; Schuler & Green, 2001). The induction of apoptosis by P53 in response to cellular stress in precancerous lesions can remove potentially dangerous cells, thereby preventing tumor growth. Deregulation of this death process results in unchecked cell proliferation, development and progression of cancer, and cancer resistance to drug therapies. Therefore, deregulation of apoptosis is considered one of the hallmarks of cancer (Fulda, 2009; Hanahan & Weinberg, 2011; Plati et al., 2008). For these reasons, therapeutic strategies targeting molecules involved in apoptotic resistance are a valid way to restore the sensitivity of cancer cells to apoptosis and overcome the ineffectiveness of treatments (Fulda, 2015; Giménez‐Bonafé et al., 2009). Since the mechanisms by which P53 fights inflammation are not yet fully understood (Kubra et al., 2020), and due to the importance of apoptosis in the pathogenesis of periodontal diseases and cancer, further molecular works are required to establish the mechanisms underlying P53's anti‐inflammatory role in human tissues. Furthermore, clinical studies with a large sample size and involving more demographic information and disease severity need to be carried out to evaluate the expression of P53 in periodontal diseases.

## 5 CONCLUSION

Several reports have shown that apoptosis has an important role in inflammatory diseases, such as chronic periodontitis, and dysregulation of it can cause cancer in tissues. One of the important factors that control regulation of cell cycle is P53 protein; a tumor suppressor that can activate apoptosis. So, downregulation of P53 gene can/will decrease the apoptotic activity and increase the risk of cancer. The primary finding to emerge from this study is that no significant difference in the expression of P53 between patients with periodontitis and the control group was found. Moreover, gender effectiveness on the percentage of staining cells was not considerable.

## AUTHOR CONTRIBUTIONS

Samaneh Minabian acquired, interpreted the data, drafted the manuscript, and revised it. Shima Soleimani S. acquired, interpreted the data, and drafted the manuscript and revised it. Molook Torabi designed the study, acquired and interpreted the data, and reviewed the manuscript. Mohammad Mohammadi designed the study, acquired and interpreted the data, and reviewed the manuscript. Hadi Ranjbar analyzed the data and revised it critically for important intellectual content.

## CONFLICT OF INTEREST

The authors declare that they have no conflict of interest.

## ETHICS STATEMENT

All procedures performed in studies involving human participants were in accordance with the ethical standards of the institutional and/or national research committee and with the 1964 Helsinki declaration and its later amendments or comparable ethical standards.

## Data Availability

The data that support the findings of this study are available from the corresponding author upon reasonable request.
